# Supplementary material for: Structural Modifications and Novel Protein-Binding Sites in Pre-miR-675—Explaining Its Regulatory Mechanism in Carcinogenesis
Source: Noncoding RNA. 2023 Aug 10;9(4):45. doi: 10.3390/ncrna9040045 (PMC10457854; doi:10.3390/ncrna9040045)
Supplement: Supplementary file 1 [file ncrna-09-00045-s001.zip › Figure S2.pdf]

A

H19_Exon1='.((((.(((..((((((((....(((...((....((...((.....))...))....))...)))..))).))))).)))....(((((.((.(((((...((((((..(((....(((.(((.((.((((((....))))))...)).))).)))...)))..))))))...((((......))))......))))))).)))))..))))........'
miR-675='(((((((.((((.((((.((((((..((((((..........))))))..)))))).)))))))).)))))))'
cofold='......,((((((((((..((((((((.((((..((((.{((..((...,,...,...))..}))........(((((....)))))...{((.((.....((((......((((.(((((..((((((.(((((((((({(({,...((((((((((......))))..))))))(((......)))))).))))))))))..))))))..((((........&.)))).))))).))))......))))....)).)))))))..)))).))))))))....)))...))))))),'

pairing_conservation

'U----U*PP++PPPPPP--++++PPP+U+PP+UU+PP+UxPPUU++U--**U--*UUUPPUUxPP-UU---UPPPPPU---++++P---p+P-+P----U++PP----UUPPP+U++PPPU-PP+PP+-PPPPP++++PpPPp*UUUPP+PPP+PPPUUU---++PP--PP+++PPPPUUUUUPPPP++U++++PPPPPP-UPPPPP+U-PPP+UUUUUUUU&-PPPP-P+PPP-+PPP-U----PP++----PPU+++++++UUPPPP-P++PPPPP-U--PPP---+PPPPPP*'

Legend: * = unknown, U = conserved unpaired, + = unpaired -> paired, x = unpaired -> pseudoknot P = conserved paired, p = paired -> pseudoknot, - = paired -> unpaired S = conserved pseudoknot, s = pseudoknot -> paired, _ = pseudoknot -> unpaired

B

H19_Exon2='.........(((..(((.((((((((((..((..(((((....(((...(((((.((.(((.((..((((..........)))).))))).)))))))...))).)).))).)).)))))))))).)))..)))(((((((.((((.((((.((((((..((((((..........))))))..)))))).)))))))).)))))))'
miR-675='(((((((.((((.((((.((((((..((((((..........))))))..)))))).)))))))).)))))))'
cofold='.........(((..(((.((((((((((..((..(((((....(((...(((((.((.(((.((..((((..........)))).))))).)))))))...))).)).))).)).)))))))))).)))..)))&(((((((.((((.((((.((((((..((((((..........))))))..)))))).)))))))).)))))))'

pairing_conservation

UUUUUUUUUPPPUUPPPUPPPPPPPPPPUUPPUUPPPPPUUUUPPPUUUPPPPPUPPUPPPUPPUUPPPPUUUUUUUUUPPPPUPPPPPUPPPPPPPPUUUPPPUUPPUPPPUPPUPPPPPPPPPPUPPPUUPPP&PPPPPPPUPPPPUPPPPUPPPPPPUUPPPPPPUUUUUUUUUUPPPPPPUUPPPPPPUPPPPPPPPPUPPPPPPP'

Legend: * = unknown, U = conserved unpaired, + = unpaired -> paired, x = unpaired -> pseudoknot P = conserved paired, p = paired -> pseudoknot, - = paired -> unpaired S = conserved pseudoknot, s = pseudoknot -> paired, _ = pseudoknot -> unpaired

C

H19_Exon3='..............((((((....))((((((((....)))))))).(((((......))))).((((.........)))).(((((....))))).)))))..........'
mir-675='(((((((.((((.((((.((((((..((((((..........))))))..)))))).)))))))).)))))))'
cofold='....((...((((((((((((((((((((((({....})))))))).........(((.(((((....((((.(((..(((((((..((..((((,,(((............&....))))))).)).)))))))))).)))))))).)))..)))))).)))).)))),..,))))...)).'

pairing_conservation

'UUUU++UUU+++++PPPPPPP++++PPPPPPPPPpUUUUpPPPPPPPU-----UUU+++-PPPP+----++++U+++U-PPP+PPP--++UUPPPP**PPP--UUUUUUUUUU&----PPP+PPPPUPP--+PPPPPP++P-PPPP+++++U+++U-PPPPP+UPPPP-P+PP*--*PP+P---PP-'

Legend: * = unknown, U = conserved unpaired, + = unpaired -> paired, x = unpaired -> pseudoknot P = conserved paired, p = paired -> pseudoknot, - = paired -> unpaired S = conserved pseudoknot, s = pseudoknot -> paired, _ = pseudoknot -> unpaired

D

H19_Exon4='.....((((((((((..((((......(((.((....((((((.((....(((...((.((....)).)).....((((((..((.....((((....))))....))..))))).))..(((((.....)))))......((((((...((((........))))))))))..................................)))....)).))))))....)).)))......)))))))))).......((((((((.((((((((((((((...((...((......(((.((((........)))))))).....)).)).))))))))))))...))).)))))))))).(((..(((((...(((..(((((.......(((....))).((((((((((((((............))))))..((((((((..(((.(((.(((...)))..)))...)))..))))))))(((((((((((..........)))))).((......)).)))))..............))))))))).........((((((((((....)))))))))).)))))..)))....)))))..)))))))..'
miR-675='(((((((.((((.((((.((((((..((((((..........))))))..)))))).)))))))).)))))))'
cofold='.....((((((((((..((((......(((.((....((((((.((....(((...((.((....)).)).....((((((..((.....((((....))))....))..))))).))..(((((.....)))))......((((((...((((........))))))))))..................................)))....)).))))))....)).)))......)))))))))).......((((((((.((((((((((((((...((...((......(((.((((........)))))))).....)).)).))))))))))))...))).)))))))))).(((..(((((...(((..(((((.......(((....))).((((((((((((((............))))))..((((((((..(((.(((.(((...)))..)))...)))..))))))))(((((((((((..........)))))).((......)).)))))..............))))))))).........((((((((((....)))))))))).)))))..)))....)))))..)))))))..&(((((((.((((.((((.((((((..((((((..........))))))..)))))).)))))))).)))))))'

pairing_conservation

'UUUUUPPPPPPPPPPUUPPPPUUUUUUPPPUPPUUUUPPPPPPUPPUUUUPPPUUUPPUPPUUUUPPUPPUUUUUPPPPPPPUUPPUUUUUPPPPUUUUPPPPUUUUPPUUPPPPPPUPPUUPPPPPUUUUPPPPPUUUUUUPPPPPPPUUUPPPPPUUUUUUUUPPPPPPPPPPPPUUUUUUUUUUUUUUUUUUUUUUUUUUUUUUUUUUUPPUUUUPPUPPPPPPPUUUUPPUPPPPUUUUUPPPPPPPPPPPPUUUUUUPPPPPPPPPPUPPPPPPPPPPPPPPPPUUUPPUUUPPUUUUUUPPPUPPPPUUUUUUUUPPPPPPPPUUUUUPPUPPUPPPPPPPPPPPPPUUUPPPUPPPPPPPPPPUPPPUUPPPPPUUPPPUUPPPPPUUUUUUUPPPUUUUPPPUPPPPPPPPPPPPPPPUUUUUUUUUUUPPPPPPUUPPPPPPPPPPUUPPPUPPPUPPPUUPPPUUPPPUUUPPPUUPPPPPPPPPPPPPPPPPPPPPUUUUUUUUUPPPPPPPUPPUUUUUPPUPPPPPPUUUUUUUUUUUUUUPPPPPPPPPUUUUUUUUUPPPPPPPPPPUUUUPPPPPPPPPPPUPPPPPPUUPPPUUUUPPPPPPUUPPPPPPPUU&PPPPPPPUPPPPUPPPPUPPPPPPUUPPPPPPUUUUUUUUUUPPPPPPUUPPPPPPUPPPPPPPPPUPPPPPPP'

Legend: * = unknown, U = conserved unpaired, + = unpaired -> paired, x = unpaired -> pseudoknot P = conserved paired, p = paired -> pseudoknot, - = paired -> unpaired S = conserved pseudoknot, s = pseudoknot -> paired, _ = pseudoknot -> unpaired
