# Supplementary figures and images for: Structural Modifications and Novel Protein-Binding Sites in Pre-miR-675—Explaining Its Regulatory Mechanism in Carcinogenesis
Source: Noncoding RNA. 2023 Aug 10;9(4):45. doi: 10.3390/ncrna9040045 (PMC10457854; doi:10.3390/ncrna9040045)

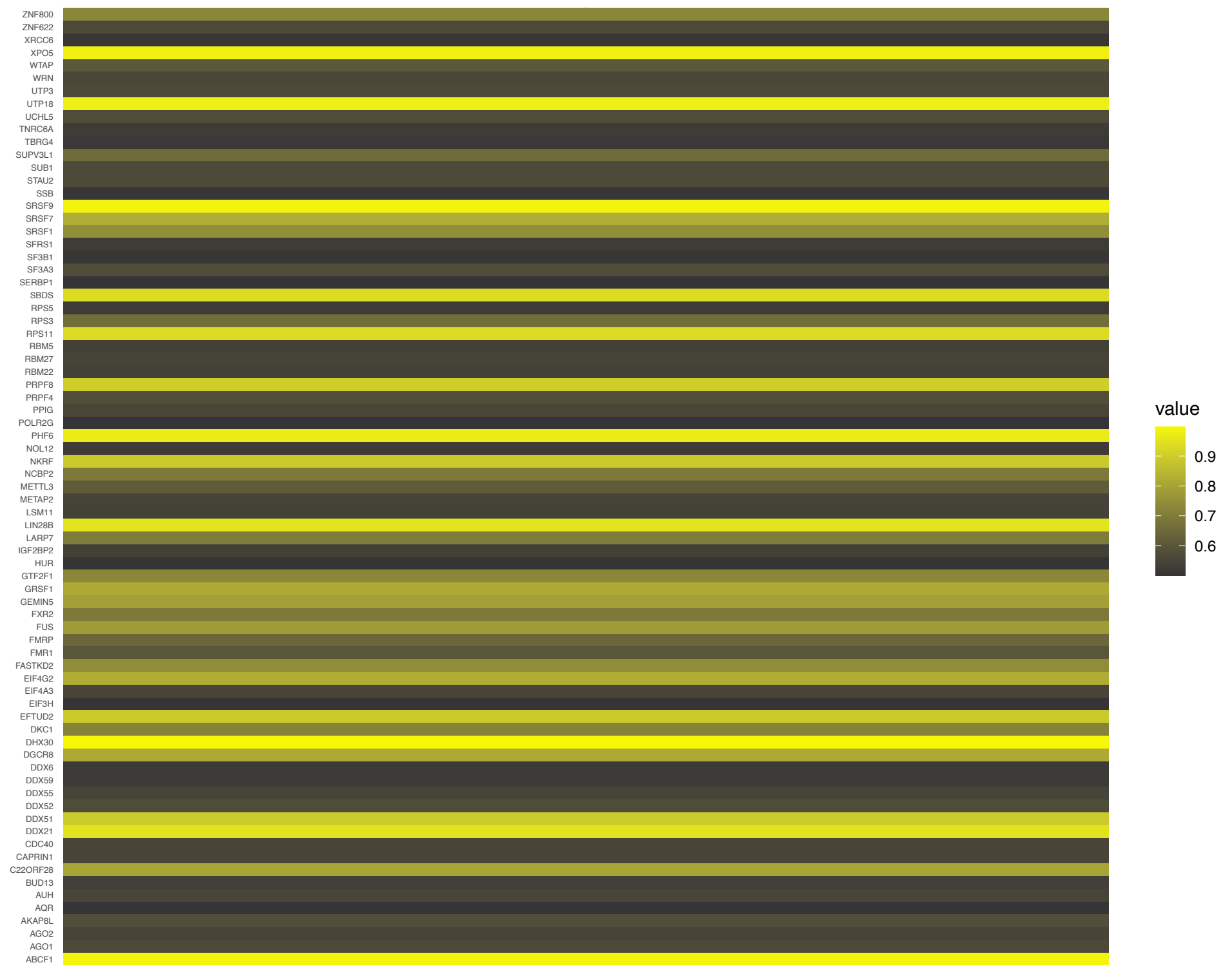

Supplement: Supplementary file 1 [file ncrna-09-00045-s001.zip › Figure S1.pdf]

A

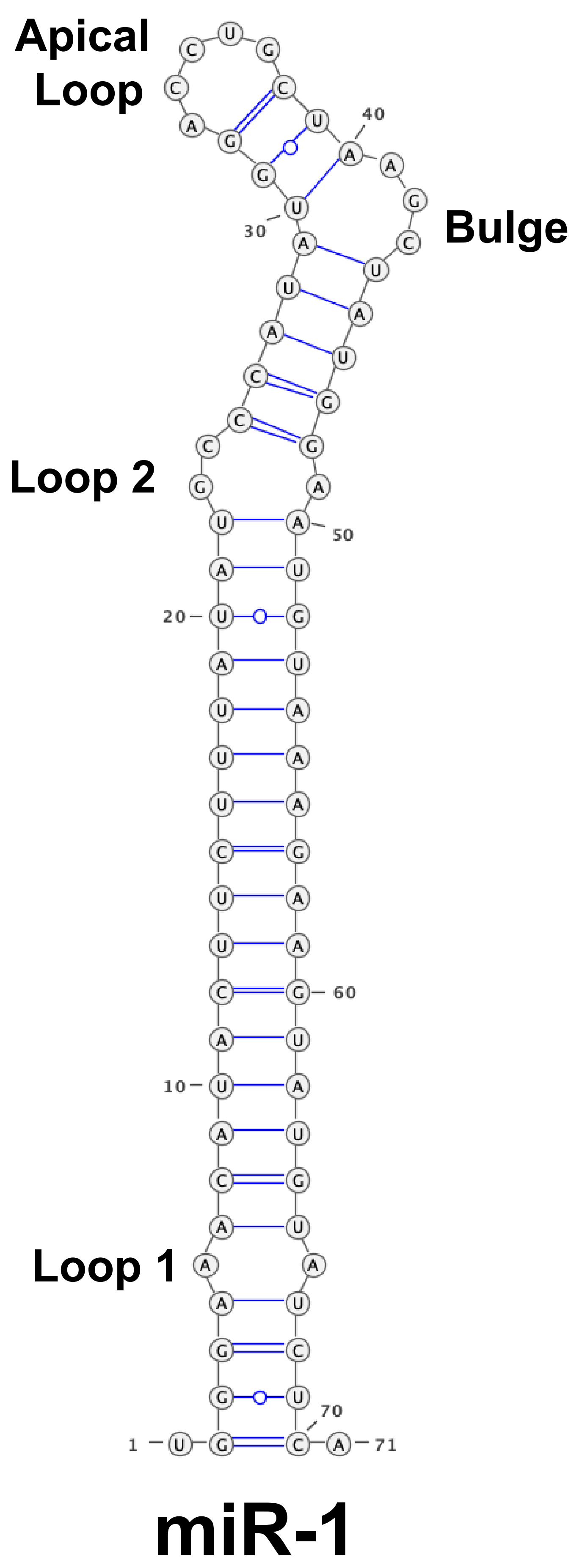

B

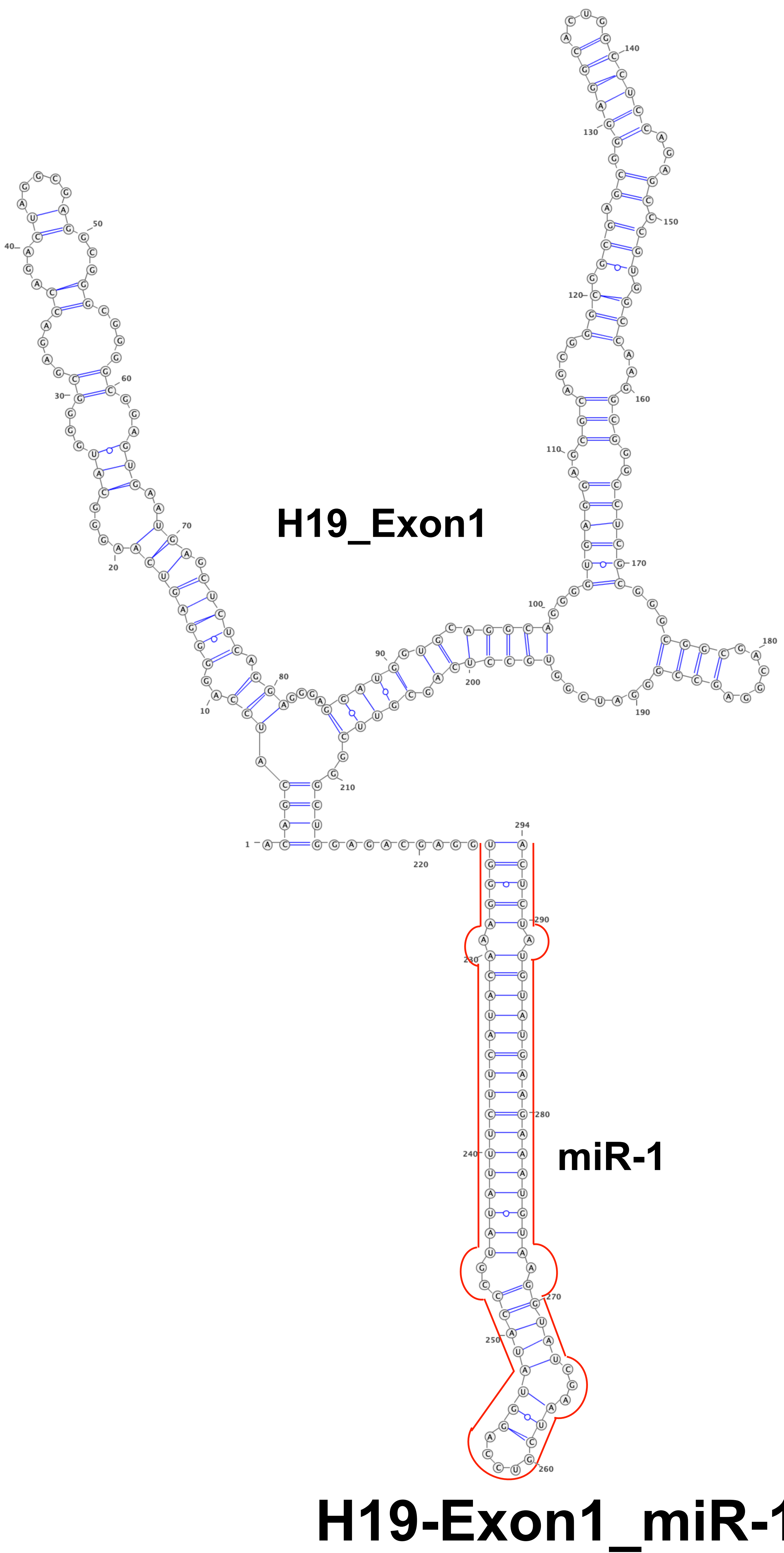

C

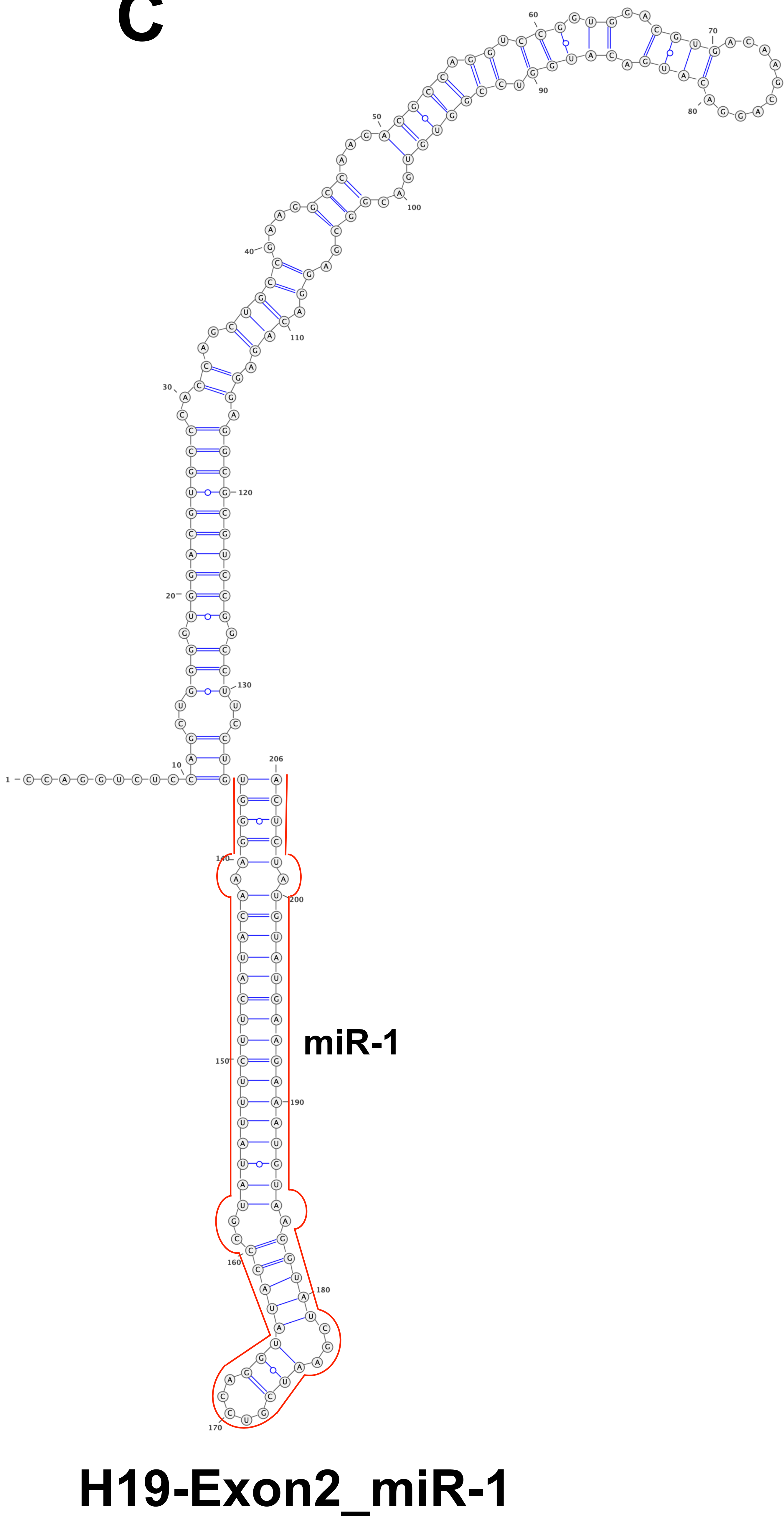

D

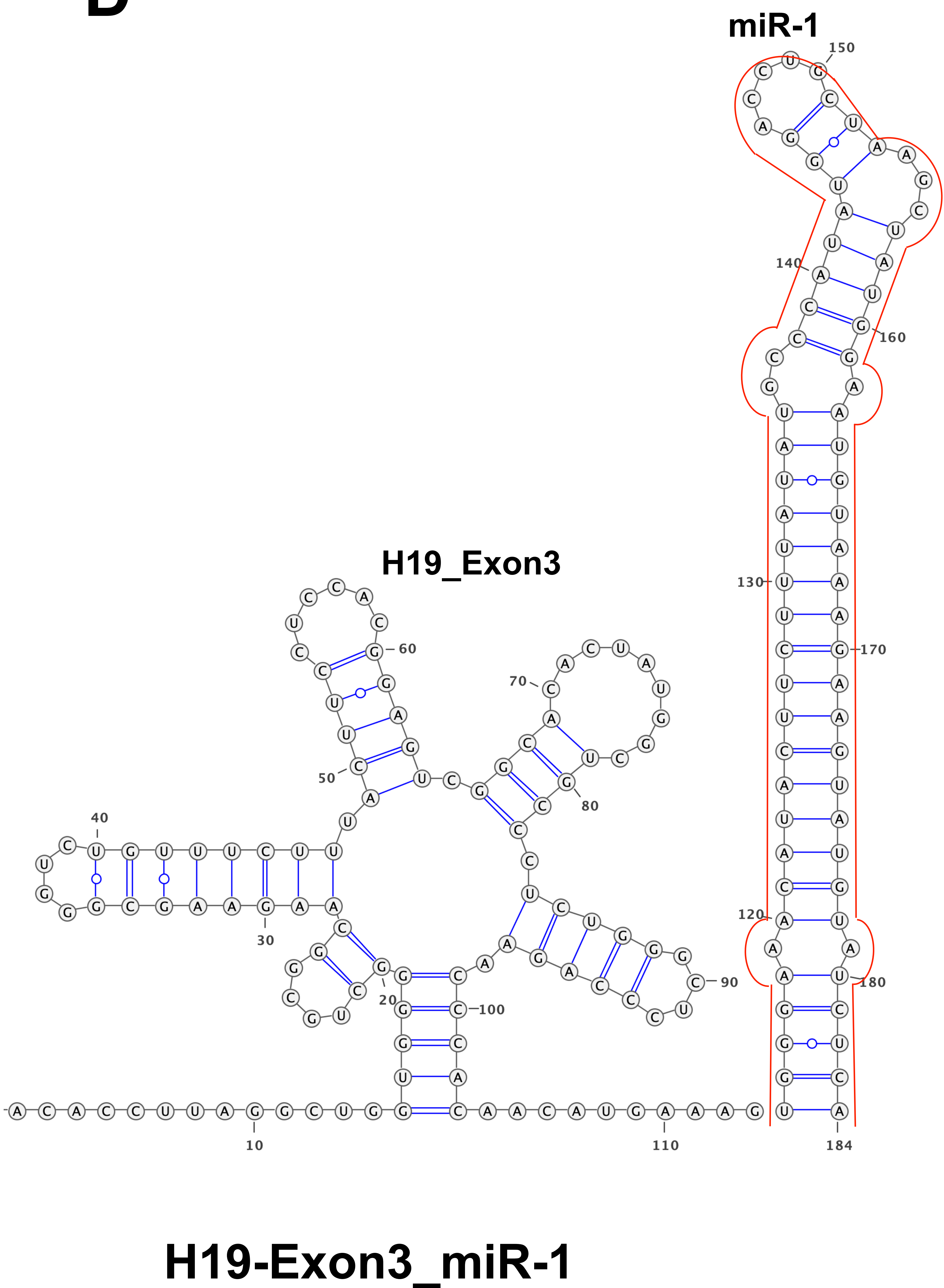

Supplement: Supplementary file 1 [file ncrna-09-00045-s001.zip › Figure S3.pdf]
