# Supplementary material for: Structural Modifications and Novel Protein-Binding Sites in Pre-miR-675—Explaining Its Regulatory Mechanism in Carcinogenesis
Source: Noncoding RNA. 2023 Aug 10;9(4):45. doi: 10.3390/ncrna9040045 (PMC10457854; doi:10.3390/ncrna9040045)
Supplement: Supplementary file 1 [file Figure S4.pdf › Figure S4.pdf]

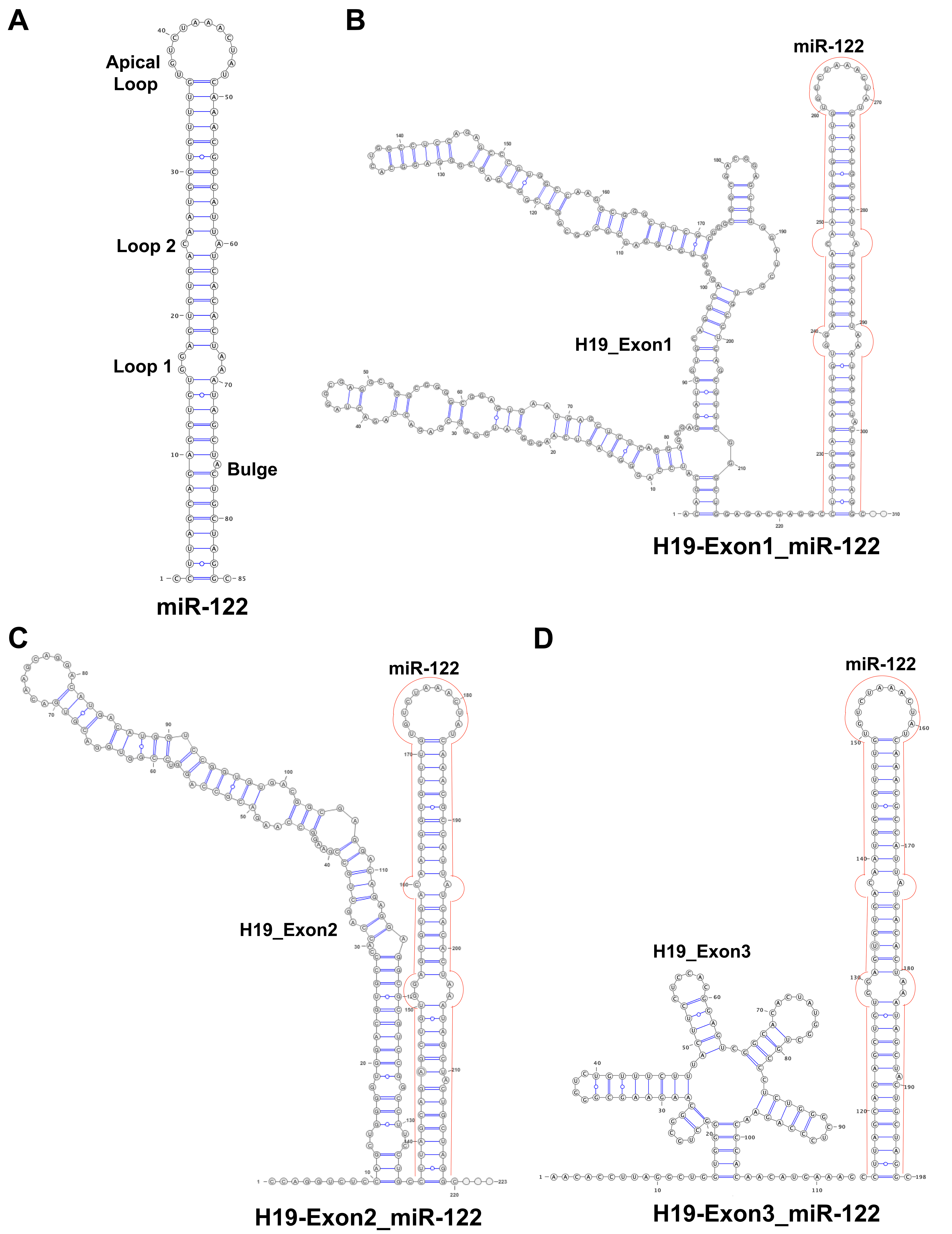
A
Apical Loop
Loop 2
Loop 1
Bulge
miR-122
B
miR-122
H19_Exon1
H19-Exon1_miR-122
C
miR-122
H19_Exon2
H19-Exon2_miR-122
D
miR-122
H19_Exon3
H19-Exon3_miR-122
